# Explaining RF induced current patterns on implantable medical devices during MRI using the transfer matrix

**DOI:** 10.1002/mp.14225

**Published:** 2020-11-28

**Authors:** Janot P. Tokaya, Cornelis A. T. van den Berg, Peter R. Luijten, Alexander J. E. Raaijmakers

**Affiliations:** ^1^ Department of Radiotherapy University Medical Center Utrecht P.O. Box 85500 Utrecht 3508 GA Netherlands; ^2^ Department of Radiology University Medical Center Utrecht P.O. Box 85500 Utrecht 3508 GA Netherlands; ^3^ Department of Biomedical Engineering Eindhoven University of Technology Eindhoven

**Keywords:** EM Simulations, implantable medical device, INduced currents, RF heating, safety, transfer matrix

## Abstract

**Purpose:**

In this work a simulation study is performed to gain insights in the patterns of induced radiofrequency (RF) currents for various implant‐like structures at 1.5 T. The previously introduced transfer matrix (TM) is used to determine why certain current patterns have a tendency to naturally occur. This can benefit current safety assessment techniques and may enable the identification of critical exposure conditions.

**Theory and Methods:**

The induced current on an elongated implant can be determined by multiplication of the incident electric field along the implant with its TM. The eigenmode spectrum of the TMs for various lengths and various types of implants are determined. The eigenvector with the highest eigenvalue describes the incident electric field pattern that induces the highest current which in turn will lead to highest heating. Subsequently, a statistical probability analysis is performed using a wide range of potential incident electric field distributions in a representative human subject model during a 1.5 T MR exam which are determined by means of electromagnetic FDTD simulations. These incident electric field distributions and the resulting induced current patterns are projected onto eigenvectors of the TM to determine which eigenmodes of the implant dominate the current patterns.

**Results:**

The eigenvectors of the TM of bare and insulated wires resemble sinusoidal harmonics of a string fixed at both ends similar to the natural‐current distribution on thin antennas(1). The currents on implants shorter than 20 cm are generally dominated by the first harmonic (similar to half a sine wave). This is firstly because for these implant lengths (relative to the RF wavelength), the first eigenvalue is more than three times bigger than the second showing the ability of an implant to accommodate one eigenmode better than another. Secondly, the incident electric fields have a high likelihood (≳95,7%) to project predominantly on this first eigenmode.

**Conclusion:**

The eigenmode spectrum of the TM of an implant provides insight into the expected shape of induced current distributions and worst‐case exposure conditions. For short implants, the first eigenvector is dominant. In addition, realistic incident electric field distributions project more heavily on this eigenvector. Both effects together cause significant currents to always resemble the dominant eigenmode of the TM for short implants at 1.5 T.

## 1 Introduction

Medical implants in patients undergoing MRI investigations can pose a safety risk. One of the main safety risks is the hazardous interaction of the implant with the transmit RF field of the MRI system. Conductive structures (like metallic implants) when exposed to the RF electric field component of the RF field tangential to the implant will accommodate currents.^1^ The currents cause charge accumulation resulting potentially into sharply peaked electric fields and local tissue heating predominantly occurring at lead tips.^2^ Especially elongated implants like guide wires,^2^ implanted pacemakers leads,^3^ ECGs,^4^ and deep brain stimulators^5^, ^6^ have shown a capacity to pick‐up and focus energy from RF electric fields. Temperature rises of up to 48°C and 20°C degrees have been found in phantom^7^ and pig^3^ experiments for guide wires and pacemaker leads respectively.

This tip heating can be calculated from the incident RF electric field with an implant characteristic called the transfer function(TF).^8^ The TF describes the contribution of a localized incident electric field at a certain position along the length of the implant to the scattered field at the tip. Thus, given a distribution of the incident electric field along the implant the scattered field at the tip can be calculated. Recently, the concept of the transfer function has been extended to the transfer matrix(TM).^9^ The TM computes the entire induced current profile over the implant from of an incident electric field along the length of the implant. This current can subsequently be used to calculate tip heating.^10^, ^11^

The extension of the TF to the TM was originally developed for MR based TF determination.^9^ In this work we will show that the TM also provides information about the shape of the current patterns that occur on implants and on worst case electric field exposure conditions. It will be shown that naturally occurring current patterns can be determined from the eigenmode spectrum of the TM of an implant because they correspond to the eigenmodes with the highest eigenvalues.

Firstly, the TMs of various elongated implant structures will be determined through electromagnetic simulations. Subsequently, the eigenmode spectrum of these TMs is calculated. Secondly, realistic incident electric field distributions inside a representative patient model, which has been extensively researched,^12^, ^13^, ^14^ are determined with simulations. These electric fields are used to compute potential currents in various implants using their TMs. The currents are subsequently evaluated in a probabilistic approach to determine what shapes of current distributions on the implant are most likely induced. It is shown that the TM of an implant provides its intrinsically supported current patterns which can help to identify hazardous and safe exposure conditions and provides insights into the resonating behavior of implants.

## 2 Materials and Methods

### 2.A Determination of the TM

The TM, $\bar{\bar{M}}$, relates the induced current pattern over the implant (described by vector $\bar{I}$) to the incident electric field along the implant (described by vector $\bar{E}$) that caused it, through:

(1) $$\bar{I}=\bar{\bar{M}}\;\bar{E}.$$

The vectors $\bar{I}$ and $\bar{E}$ are distributions discretized to 5 mm resolution of respectively the induced current in the implant and the tangential component of the electric field incident on the implant. This corresponds to the resolution with which the TM is resolved. Bare and partially insulated wires of various lengths are investigated. Their TMs are computed from electromagnetic simulations(Sim4Life, ZMT, Zurich, Switzerland) where currents are induced in the various implants by electric field sources^9^ that generate a constant incident field within a box of 5mm width and no incident field outside it (referred to as ‘plane wave sources’ in the software package). The simplest implant is a bare wire with a 2.5 mm diameter, for which the TM is determined for wire lengths of 10cm, 20cm, 30cm, and 40cm with a resolution corresponding to the width of the plane wave sources. The TM of the 20 cm wire is also determined for a situation where one end of the wire is attached to a cube of perfect electric conductive material with 4 cm edges intended to mimic an implantable pulse generator (IPG). A third 20 cm wire is composed of an 8 cm insulated region and a 12 cm bare region to explore the effect of an impedance transition. Another set of wires for which the TM is determined is insulated except for a small bare part of 1cm at both ends. Also, these wires were studied for a wire length of 10cm, 20cm, 30cm, and 40cm. An overview of all investigated structures is shown in Table I. All structures are gridded with a 0.2 mm resolution perpendicular to the long axis of the implant and 0.5 mm resolution along the long axis.

**Table I:**
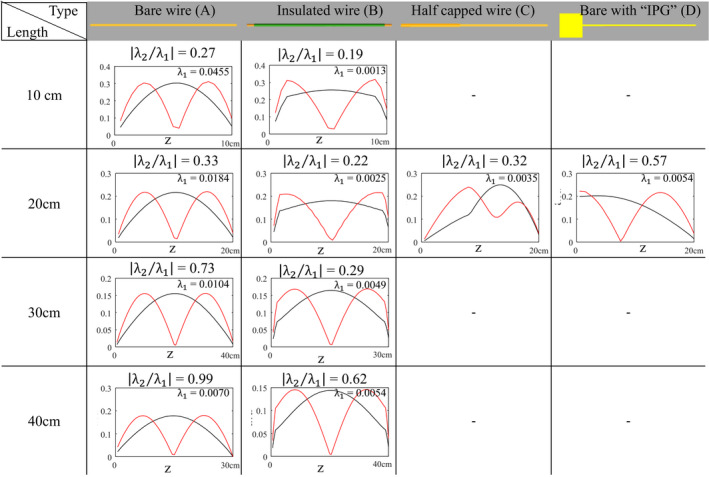
The first and second eigenvector of the TMs of the various investigated structures are shown as black and red lines respectively. The fraction between the corresponding 1<sup>st</sup> and 2<sup>nd</sup> eigenvalue, i.e. <span class="ieqmath"><span altimg="urn:x‐wiley:00942405:media:mp14225:mp14225‐math‐0007" wiley:location="equation/mp14225‐math‐0007.png" data‐pi‐after="7" xml:id="math7" class="math" style="display:None"><msub><mi mathvariant="bold‐italic">λ</mi><mn>1</mn></msub></span><img align="middle" class="Origformula" xml:id="mathImage7" onclick="window.parent.EditEquation(this)" src="OPS/20200521072451748/ProofLink2/htmlequation/mp14225‐math‐0007.png"></img></span> and <span class="ieqmath"><span altimg="urn:x‐wiley:00942405:media:mp14225:mp14225‐math‐0008" wiley:location="equation/mp14225‐math‐0008.png" data‐pi‐after="8" xml:id="math8" class="math" style="display:None"><msub><mi mathvariant="bold‐italic">λ</mi><mn>2</mn></msub></span><img align="middle" class="Origformula" xml:id="mathImage8" onclick="window.parent.EditEquation(this)" src="OPS/20200521072451748/ProofLink2/htmlequation/mp14225‐math‐0008.png"></img></span>, increases with increasing implant length. This shows that the current pattern on longer wires will start to become a superposition of multiple eigenvectors, whereas the current pattern on the shorter implants is essentially dominated by the first eigenvector. Note that for the lengths displayed here the first eigenvector always has one maximum. For longer implants (or higher frequencies) the eigenvector with the highest eigenvalue can also have multiple maxima.

Note that the simulation method of subsequently repositioned localized electric field excitations for TM determination used here is equivalent to the piecewise excitation method^8^, ^15^ used for transfer function determination with the distinction that the entire induced current distribution and not only the tip field is monitored.

The conductive parts of the wires are made of copper with a conductivity (at 64MHz) of 5.8·10^7^ S/m. The 0.5mm thick insulation layer has a relative permittivity of 3 and is nonconductive. These generic structures were used in this exploration because their TMs will vary significantly and show different responses to an incident electric field. Furthermore, the bare and insulated wires of 20cm length have known properties and have been used in other works.^8^, ^16^, ^17^ All the structures were simulated in a saline solution (relative permittivity of 78 and a conductivity of 0.47 S/m) that filled the entire computational domain. These properties correspond to the medium described in test standards.^18^

Once the TM of an implant has been simulated, the eigenvalues and eigenvectors of this matrix of size NxN are determined with MATLAB (MathWorks, Natick, MA) using *LU* factorization. These eigenvectors give the characteristic eigen current modes in the implant. One can always decompose (part of) the incident electric field in the first k eigenvectors (corresponding to the k largest eigenvalues) of the TM. Therefore,

(2) $$\bar{I}=\bar{\bar{M\;}}\bar{E}=\bar{\bar{M}}\alpha_{1}\bar{v_{1}}+\alpha_{2}\bar{v_{2}}+\ldots +\alpha_{k}\bar{v_{k}}+\bar{E_{r}}.$$

Here $v_{i}$ stands for the $i^{\mathrm{th}}$ eigenvector of $\bar{\bar{M}}$. $\alpha_{i}$ is the coefficient in the eigenvector decomposition. It is computed by taking the normalized inner product of the corresponding eigenvector with the incident field, i.e. $\bar{v_{i}}\cdot \bar{E_{\mathrm{inc}}}/∥\bar{E_{\mathrm{inc}}}∥$. $\bar{E_{r}}$ is the part of the electric field that is not decomposable into the first k eigenvectors of the TM and is generally extremely small after inclusion of only a few eigenvectors. Hence,

(3) $$\bar{I}\approx \lambda_{1}\alpha_{1}\bar{v_{1}}+\lambda_{2}\alpha_{2}\bar{v_{2}}+\ldots +\lambda_{k}\alpha_{k}\bar{v_{k}}.$$

It is clear that particularly the eigenvector with the highest eigenvalue has the potential to manifest itself as induced current pattern when it is ‘excited’ because the contributions of the various eigenvectors are weighted by the corresponding eigenvalue. This occurs when the inner product between the electric field and this eigenvector is large. The first two eigenvectors of the various structures are shown in Table I.

### 2.B Determination of possible electric field exposures

**Fig. 1:**
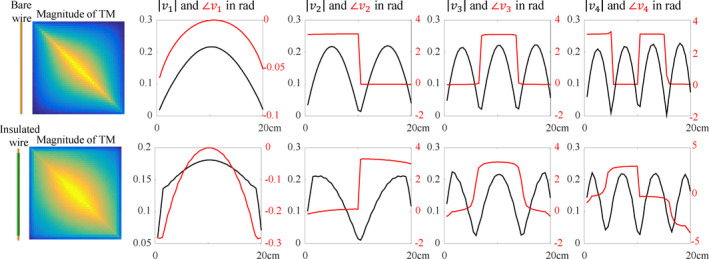
The TM of the bare (top) and insulated wire (bottom) of 20cm length together with its first four eigenvectors. The eigenvectors are sine shaped modes with increasing number of nodes on the length of the implant. For the structures investigated in this work the first eigenvector always is the mode with smallest number of nodes. This potentially changes if the length of the implants exceeds the maximal length investigated here and will depend on the ratio between the wavelength and the length of the implant. It will result in a different ordering of the modes, but not in an alteration of their overall appearance. [Color figure can be viewed at wileyonlinelibrary.com]

Another FDTD electromagnetic simulation is performed to determine realistic incident electric field distributions. These distributions are determined from a simulation of the “Duke” model from the Virtual Family^19^ positioned for a cardiac MRI in a 1.5 T (or 64MHz) highpass birdcage coil, driven in quadrature. The simulation setup is shown in Fig. 1. The birdcage coil has 16 rungs, a 35.2 cm coil radius and 42 cm rung length. The RF shield has a 37.2 cm radius, 70 cm length and is composed of perfect electric conductor. The Duke model was resolved in 2 mm isotropic grid. The simulation time is set to 100 periods leading to a final power balance of 100.53%.

From the electric field distribution inside the Duke model, that is predominantly z‐oriented, the potential electric field exposures along the implant are extracted. For this purpose, the complete set of possible positions of the implant within the model is evaluated and for each position the incident electric field *E_z_* along the implant is extracted. To speed up this process the electric field inside the Duke model is linearly interpolated to a 2 × 2 × 5 mm resolution. The resolution in the z‐direction corresponds to the resolution of the TM, i.e. the width of the plane wave boxes used in the piecewise excitation method. For all positions, the wires are assumed to be z‐oriented because this configuration will for the application presented here generally lead to worst case exposure conditions and are furthermore most likely to occur for linear implants. In some regions other components of the electric field distribution inside the “Duke” model will also be significant, but since only z‐directed implants are considered in the analyses presented here these components will not induce currents. All connected $E_{z}$ field distributions of 10 cm, 20 cm, 30 cm, and 40 cm length along the z‐axis inside the human model are extracted from the interpolated electric field, i.e. the complex $E_{z}$ values inside, respectively, all 20, 40, 60, and 80 voxel stacks in the z‐direction within the Duke model for the 10 cm, 20 cm, 30 cm, and 40 cm wire create the distribution of $E_{\mathrm{inc}}$ fields. The result is a collection of millions of possible incident E‐fields along an implant. Despite being obtained from a single simulation, this large number is assumed to be a representative collection of potential electric field exposures. The discussion section will present a verification of this assumption.

### 2.C Determination of possible induced current patterns

The TM is applied to gain insight into the current patterns that can be excited on an implant given the collection of potential incident electric field exposures. Multiplication of the transfer matrix with all possible incident $\bar{E_{z}}$ distributions will result in the corresponding current patterns. Note that the highest average $\bar{E_{z}}$ does not necessarily correspond to the highest current.

Next to a direct calculation of the currents on the wires a more detailed analysis of the resulting current patterns is performed based on the eigenvectors of the TM. Firstly, all the electric field distributions are decomposed into the eigenvectors of the TM to identify modes that are ‘excited’ more heavily. The projection of eigenvector $\bar{v_{i}}$ onto a particular $\bar{E_{z}}$ distribution will be denoted with $\alpha_{i}$, i.e. $\bar{v_{i}}\cdot \bar{E_{\mathrm{inc}}}\backslash ∥\bar{E_{\mathrm{inc}}}∥$. This projection purely displays how efficient an external field can excite certain current modes and contains no information on the ability of an implant to accommodate a certain mode.

Secondly, after obtaining the induced current on the wire by multiplication of $\bar{E_{z}}$ with the TM the actual contribution of a particular eigenvector to this distribution will be denoted by $\beta_{i}$, i.e. $\bar{v_{i}}\cdot \bar{I_{\mathrm{ind}}}\backslash ∥\bar{I_{\mathrm{ind}}}∥$. In these projections the weighting due to the magnitude of the corresponding eigenvalue are incorporated. The magnitude of the eigenvalue is a quantitative measure of the ability of an implant to accommodate the corresponding eigenvector.

## 3 Results

Figure 1 shows the magnitude of the TMs of the bare and insulated wire of 20 cm length with their first four eigenvectors. These eigenvectors come in the shape of sine waves with increasing frequency similar to the natural harmonics that appear on a musical string when it is fixed on both ends. The eigenvectors of the bare and insulated wires of other lengths follow similar patterns. The eigenvectors of the TM of the partially insulated wire shows a resemblance to the harmonics on a string with variable density along the length of the wire. The TM of the wire that is attached to a conductive block has eigenvectors similar to the standing waves in a pipe with one open end (the end attached to the conductive block). This is similar to the current on a quarter wavelength grounded antenna.^20^ The endpoint of this implant, where it is attached to the IPG, acts as if it is grounded and hence open. The other end which is embedded in poorly conducting material is closed.

Table I shows that the bare and insulated implants with a length of 20 cm and shorter have a 1^st^ eigenvalue that is three or more times bigger than the 2^nd^ eigenvalue. For the 20 cm and 10 cm bare wires the second eigenvalue is only 27% and 33% of the first eigenvalue respectively. In the other structures this dominance is also found with an exception of the bare wire connected on one end to a conductive block, which has a second eigenvector that is 57% of the first. The dominant first eigenvalue shows that one specific current pattern (the eigenvector corresponding to this eigenvalue) will have the largest contribution to the overall current distribution. The dominance of certain modes depends on the length of the implant and the wavelength of the RF field in the medium and is related to resonance effects.^21^ The wavelength of electromagnetic waves in the test medium at 64MHz is 44 cm.^22^ The implants shorter than 20cm (less than half a wavelength) can therefore only accommodate a single mode (the one with a single maximum) whereas the 40cm implants are already able to accommodate higher modes as can be seen by the large magnitude of the second eigenvalue.

After the simulation of the incident electric field in the Duke model in total 2.83 million electric field exposures of 10 cm, 2.27 million electric field exposures of 20 cm, 1.85 million electric field exposures of 30 cm, and 1.53 million electric field exposures of 40 cm are extracted. When integrating the electric fields over the implant length, the highest electric potentials are 268V, 445V, 572V, and 657V for 10 cm, 20 cm 30 cm, and 40 cm length respectively after scaling the B_1_^+^_max_ of the birdcage coil to 30 μT in the center of the imaging domain for this particular simulation.

**Fig. 2:**
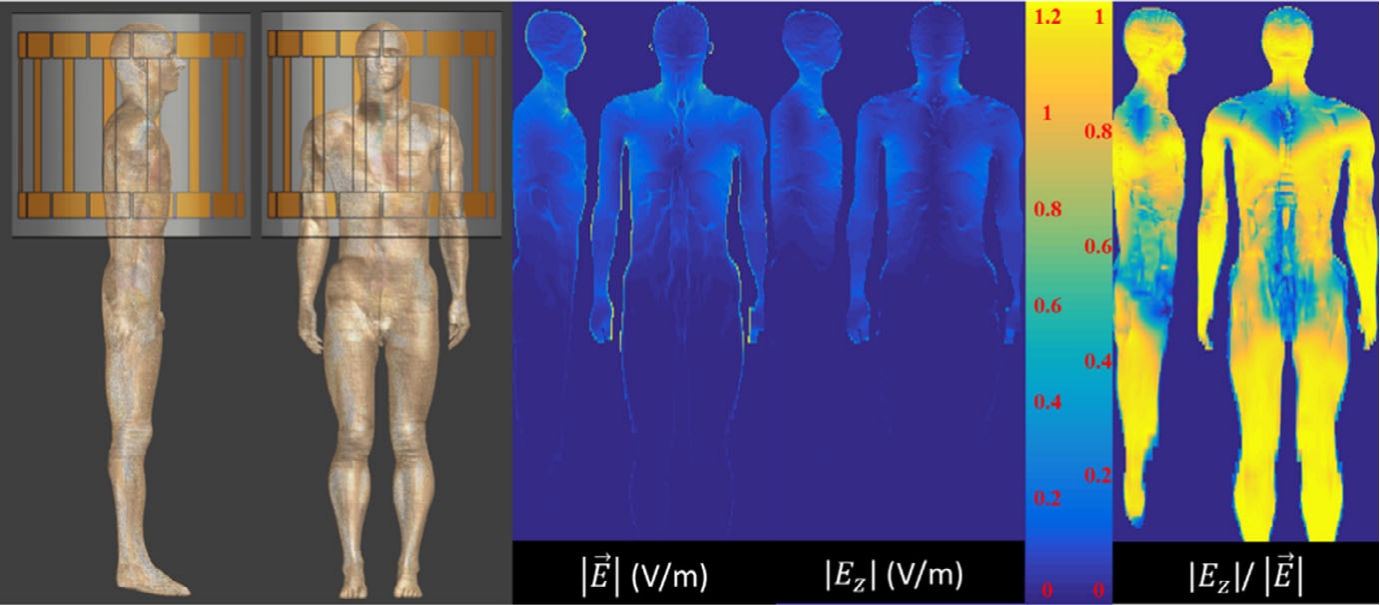
The simulation setup of the Duke model in the birdcage is shown on the left. The electric field distributions (scaled to 30 μT peak B_1_^+^), resulting from the simulations performed with this setup, show that the electric field inside the Duke model is predominantly z‐oriented. From the $E_{z}$ distribution inside the Duke model all possible 10 cm, 20 cm, 30 cm, and 40 cm distributions in the z‐direction are extracted. These distributions represent all possible electric field exposures that an implant of identical length directed along the z‐axis (often worst case) can encounter. [Color figure can be viewed at wileyonlinelibrary.com]

**Fig. 3:**
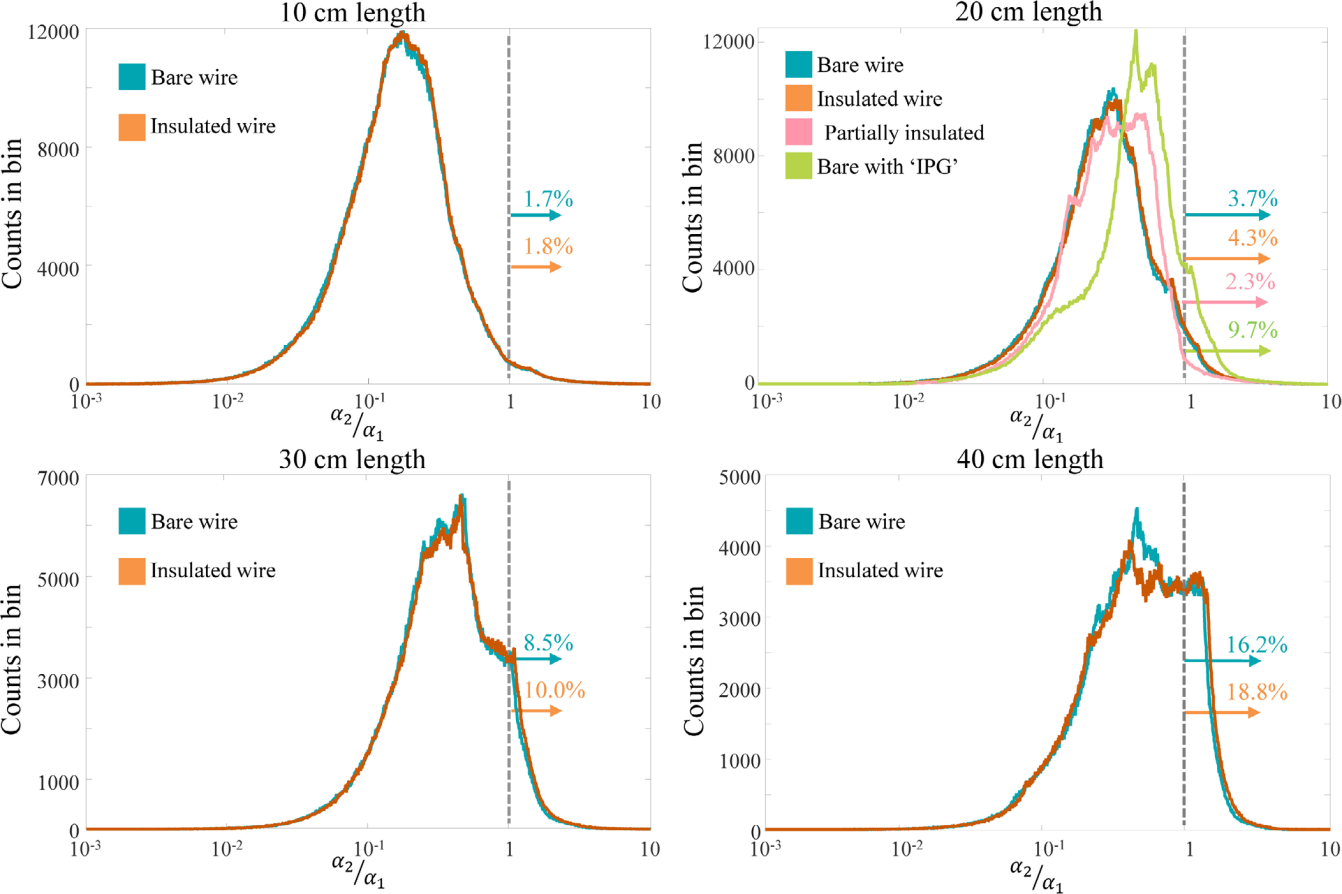
The electric field distributions of the various lengths shown in Fig. 2 are projected onto the first two eigenvectors of the TM of the various implants with the same length. Here, histograms of the fraction between both projection values are displayed. If $\alpha_{2}/\alpha_{1}$>1, the incident electric field has a larger projection onto the second eigenvector. For the wires shorter than 20cm more than 95% of the electric field distribution are more efficient in exciting the first eigenvector of the TM, with exception of the bare wire connected to a conductive block. [Color figure can be viewed at wileyonlinelibrary.com]

These electric field distributions are projected onto the first and second eigenvector of the TM of the various implants. The projections are denoted by $\alpha_{1}$ and $\alpha_{2}$. The ratio between $\alpha_{1}$ and $\alpha_{2}$ indicates how strongly an incident electric field couples to the first eigenvector with respect to the second. The distributions of these ratios for all possible electric field exposures are shown in Fig. 3 as histograms for the bare and insulated wire. Figure 3(a), for example, shows two graphs. One for the bare and one for the insulated wire, which are more or less similar. The vertical dashed line displays where the ratio $\alpha_{2}$/ $\alpha_{1}$ equals 1. The large majority of the potential incident electric field distributions results in a $\alpha_{2}$/ $\alpha_{1}$ ratio smaller or much smaller than 1 (i.e. the first eigenvector is dominant) with a maximum likelihood around $\alpha_{2}$/ $\alpha_{1}=$ 0.176 and 0.171 for the bare and insulated wire respectively. Only in 1.7 and 1.8% of the cases, for the bare and insulated wires, respectively, the projection of the incident field on the second eigenvector is larger.

Looking at all histograms, it is clear that for all wire lengths exciting the first eigenvector is more likely than exciting the second eigenvector. This likelihood goes down with increasing implant length from ca. 98% for 10 cm wires to 81% for 40 cm wires. On top of this, in the less likely situation that the incident electric field excites the second eigenvector stronger, the amplitude of that electric field distribution tends to be lower: the worst‐case electric fields that have a larger projection onto the second eigenvector (of a bare wire) compared to the first, i.e. $\alpha_{2}>\alpha_{1}$, are respectively only 73%, 47%, 39%, and 41% of the highest electric potentials in general. So even though it can happen that the second mode is excited more effectively by an incident field then this will not correspond to the worst‐case exposure.

**Fig. 4:**
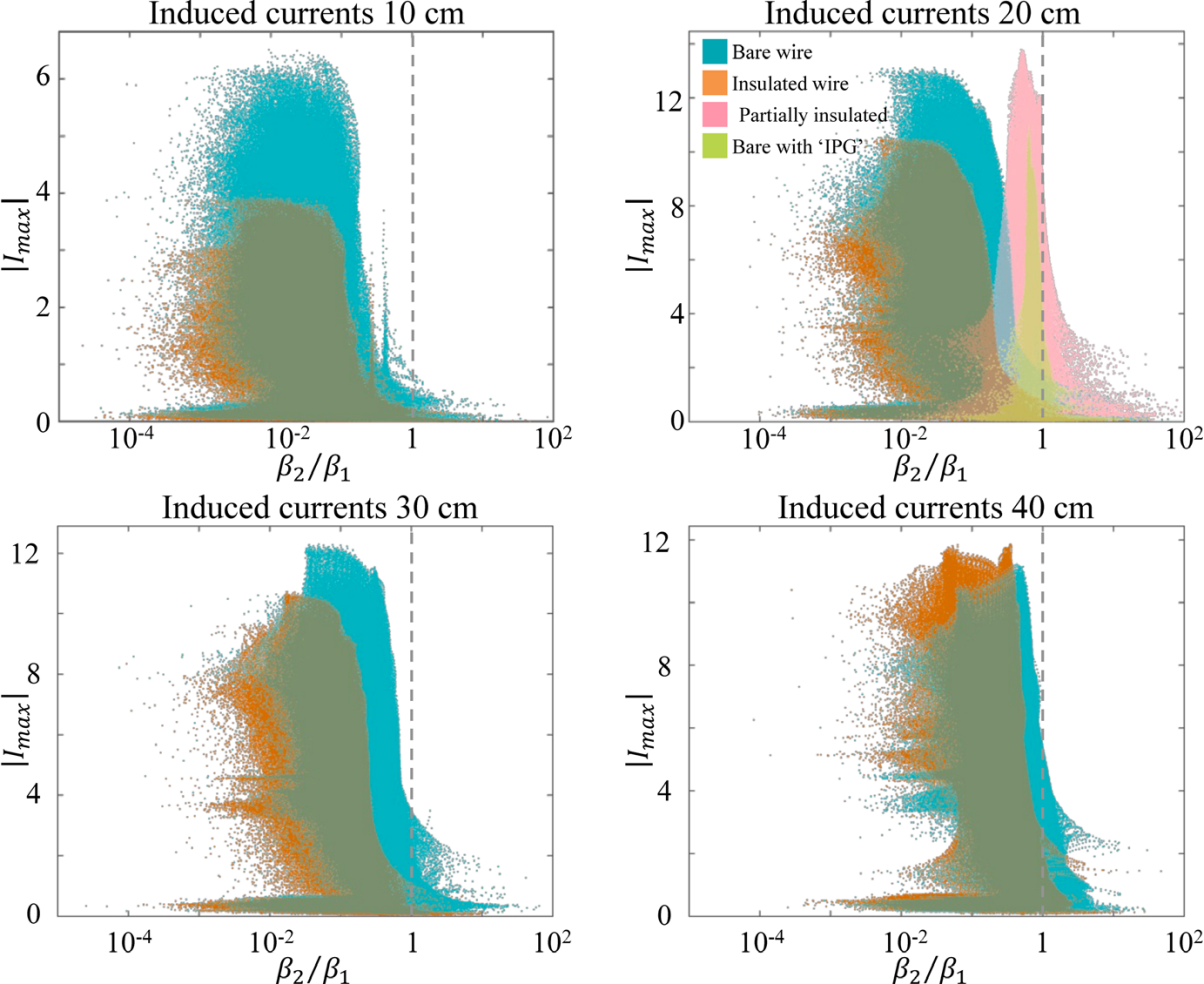
The maximum of the current distribution in the wires of various lengths plotted against the fraction between the projection of the 2^nd^ and 1^st^ eigenvector of the TM onto this distribution. When this fraction is higher than 1 the current is more similar to the second eigenvector of the TM. From these plots it is clear that the currents induced on the wires predominantly take on a pattern comparable to the first eigenvector which was already to be expected in view of Fig. 3. It is furthermore clear that when a current higher than 50% of the worst‐case current is induced the distribution will always be dominated by the first eigenvector. [Color figure can be viewed at wileyonlinelibrary.com]

Multiplication of the incident electric fields with the TM results in the total induced currents on the implant. The current distributions are, like the incident electric fields, projected onto the first two eigenvectors of the TM. These projections, denoted by $\beta_{1}$ and $\beta_{2}$, describe to which eigenvector the induced currents are more similar. These values will be influenced by both the effectiveness of the electric field in exciting a certain eigenvector and the ability of the implant to support this mode. In Fig. 4 the maximal current in the implant is displayed against $\beta_{2}/\beta_{1}$. In these scatter plots each point represents one potential incident electric field distribution (i.e. one potential implant position in our simulated Duke model). The horizontal position of each point represents the $\beta_{2}/\beta_{1}$ ratio. If $\beta_{2}/\beta_{1}<$ 1 the current distribution will be more like the first eigenvector. If $\beta_{2}/\beta_{1}>$ 1, the current will be more like the second eigenvector or contain even contributions from higher eigenvectors. The vertical position of each point represents the largest current amplitude along the wire for this incident electric field distribution. For 10 cm wires, the large majority of potential incident electric field distributions results in a current distribution along the wire that is highly similar to its first eigenvector ( $\beta_{2}/\beta_{1}$<< 1). Only a very small minority of the potential incident electric fields realizes current distributions along the implant that are more similar to the second eigenvector. However, in these situations the maximum amplitude of the induced current is rather low. From Fig. 4 it can be seen that for longer implants the induced currents start to have a larger contribution from the second eigenvector because the entire distribution is shifted toward the region where $\beta_{2}/\beta_{1}>$ 1. For all lengths, the amplitude of current distributions that are more similar to the second eigenvector are well below the maximum amplitude of induced currents in general. In addition, this situation is less likely to occur. The distribution of current maxima is on the verge of crossing the $\beta_{2}/\beta_{1}$ = 1 line for the 40 cm bare wire. This indicates that at 1.5 T wires longer than approximately 40 cm will start to have a dominant eigenvector with two current maxima.

**Fig. 5:**
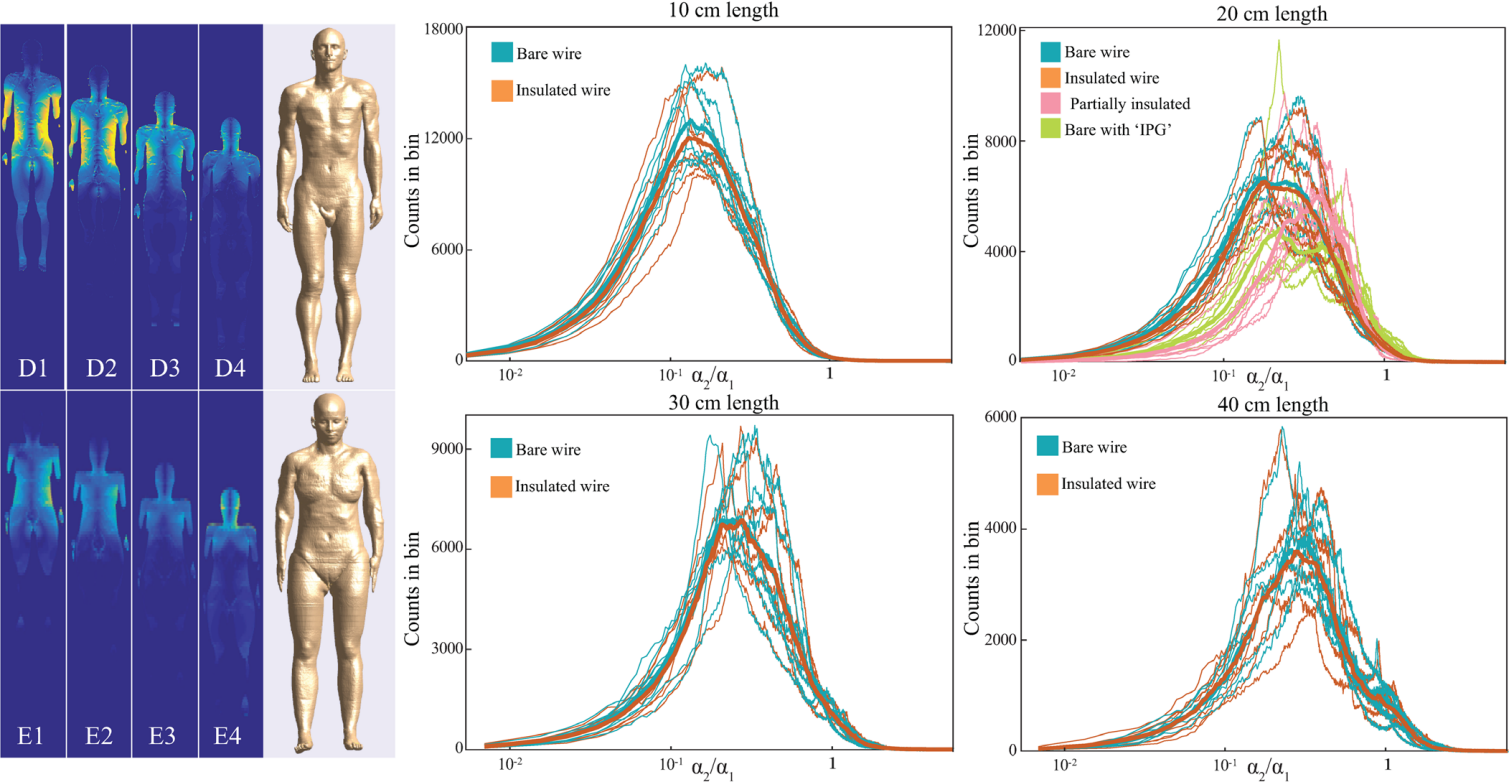
All possible incident electric field distributions from the simulated electric fields in the Duke and Ella model at various locations w.r.t. the bodycoil shown on the left are projected onto the first two eigenvectors of the TM of the various implants. Here, histograms of the fraction between both projection values are displayed. If $\alpha_{2}/\alpha_{1}$>1, the incident electric field has a larger projection onto the second eigenvector. For the wires shorter than 20cm more than 90% of the electric field distribution are more efficient in exciting the first eigenvector of the TM, with exception of the bare wire connected to a conductive block. [Color figure can be viewed at wileyonlinelibrary.com]

## 4 Discussion

Numerical simulations are performed to determine the TM of various implants. These TMs are subsequently multiplied with a large number of potential incident electric field distributions. These distributions are obtained by extracting the electric field values along the z‐directed wire for all physically possible positions inside a human model. The electric field distribution inside this model is also determined by numerical electromagnetic simulations. The resulting current distributions always tend to appear in a pattern similar to the first dominant eigenvector of the TM of this implant. This especially holds for implants shorter than 20 cm. The dominant eigenvectors are always current patterns with one maximum along the implant for the lengths and implants investigated in this work. Note that for longer implants and/or larger frequencies, it is likely that the largest eigenvector may in fact show two maxima and the second eigenvector only has one. This essentially interchanges the eigenvectors when ordered based on the magnitude of the corresponding eigenvalue. The first and second eigenvectors might both contribute significantly for longer implants. The current in the implant can always be decomposed into the eigenvectors of the TM, but generally since the eigenvalues decrease quickly in magnitude a decomposition with only the first few eigenvectors leads to an accurate approximation. It should still be verified that the dominance of one eigenmode found for the generic models investigated in this work holds for other (realistic) implants. Particularly the implant with the IPG already at a shorter length has a relatively big contribution from higher eigenmodes. The IPG was simulated as a conductive block shorted with respect to the lead, which corresponds to the situation where induced currents in the lead are guided towards the casing of the IPG. Depending on how the currents are routed in a particular implant this behavior might change which will have an effect on the eigenmode spectrum of the transfer matrix. The relative dominance of certain eigenmodes will therefore be dependent on the characteristics of the connection between the lead and the IPG.

Knowledge of the TM of an implant is a prerequisite to determine eigenvector resonances and the corresponding current distributions. If an implant with unknown characteristics is present this knowledge might be available or attainable by means of simulations^8^, ^17^ or measurements.^15^, ^23^ Next to an alternative way of understanding induced current patterns on medical implants knowing these eigenvectors could prove beneficial for RF safety assessment of implants by current mapping.^24^, ^25^, ^26^ Once the eigenvectors of the TM are known, the current patterns will be accurately resolvable in a weighted sum of a limited number of eigenvectors. Hence the entire distribution can be determined from a few sample points and therefore knowing the current in a couple of slices will be sufficient. This can speed up measurements of induced currents. Potentially, it could facilitate extrapolation of measured current distributions to outside the field of view of the MR scanner. The latter might aid for example MR‐guided catheter‐based procedures.”

The TM of the implants was determined through simulations in a background of high permittivity medium as described in safety standard.^18^, ^27^ This TM is subsequently used to determine currents induced at various locations inside the Duke model without considering potential changes in the TM due the local dielectric properties. The transfer function, and hence also the TM, is however known to be dependent on the dielectric properties of its surroundings^28^, ^29^, ^30^ and this straightforward translation will introduce an inaccuracy. The extent of this inaccuracy and the way it will influence the current distributions shown in Fig. 4 is interesting, but is beyond the scope of this paper. An explorative study was presented by Kozlov et al.^31^

In this work the electric field distribution inside a human subject is only simulated for one specific model at one specific imaging position in one specific RF coil. Extensions to more imaging positions of the Duke model and the Ella model are performed to investigate if the found results are representative for other scenarios. For these 8 simulations again, the electric field distributions for every possible z‐oriented location (in a discretized sense) are extracted and decomposed into the eigenvectors of the various investigated structures. The distributions of fractions between the first and the second coefficient in this eigenvector decomposition are displayed in Fig. 5. Here, it can be seen that there are differences in the distributions which originate from differences in the simulated electric field distributions. This also results in different fractions of the electric field distribution where the projection of the incident field on the second eigenvector is larger than the projection on the first eigenvector. This can be seen in Table II. Here the percentage of incident electric fields with a larger projection on the second eigen vector is given for the eight simulations and the various implants. Although the exact numbers are slightly different, overall the simulations lead to similar distributions and the percentages in Table II corroborate the finding that the first eigenvector is more likely to be excited than the second.

**Table II:** The percentage of incident electric fields from the various simulated distributions with a larger projection on the second eigenvector than on the first eigenvector of the TMs of the various investigated structures.

Simulation number	Bare wire of 10 cm length (%)	Insulated wire of 10 cm length (%)	Bare wire of 20 cm length (%)	Insulated wire of 20 cm length (%)	Partially insulated wire (%)	Bare wire with ‘IPG’ (%)
D1	2.6	2.7	10.3	9.1	7.2	17.5
D2	1.7	1.8	4.3	3.7	2.3	9.7
D3	3.1	3.3	10.3	9.6	7.9	18.4
D4	2.4	2.5	6.1	5.8	9.6	7.7
E1	4.0	4.2	11.9	11.2	8.4	16.8
E2	1.2	1.2	6.1	5.0	3.1	13.3
E3	3.3	3.4	11.8	10.6	6.8	22.0
E4	2.6	2.6	7.4	7.3	8.5	11.3
Average	2.6 ± 0.9	2.7 ± 1.0	8.5 ± 2.9	7.8 ± 2.7	6.7 ± 2.6	14.6 ± 4.9

	Bare wire of 30 cm length (%)	Insulated wire of 30 cm length (%)	Bare wire of 40 cm length (%)	Insulated wire of 40 cm length (%)
D1	18.3	20.2	27.9	30.5
D2	8.5	10.0	16.2	18.8
D3	17.0	18.4	24.7	25.9
D4	8.0	8.4	25.3	27.8
E1	19.6	20.6	26.4	27.7
E2	15.3	16.9	25.3	19.8
E3	19.4	20.7	24.9	26.4
E4	9.7	10.0	13.0	13.4
Average	14.5 ± 5.0	15.6 ± 5.3	23.0 ± 5.4	23.8 ± 5.8

In the letter D in the first column stands for simulations with the Duke model and E stands for simulations with the Ella model. The position of the human model moves downward 20 cm in the z‐direction with increasing simulation number. Simulation D2 corresponds with the results shown in Figs. 2, 3 and 4.

Future work should verify if the millions of incident electric field distributions extracted from these situations are representative for even more imaging positions, body models, various coils, and/or implant orientations.

## 5 Conclusion

The eigenvectors of the transfer matrix of wire‐like implants show similarities to naturally occurring current patterns on thin dipole antennas resembling sinusoidal standing wave patterns. The endpoints of these dipoles are grounded (open) when attached to a ‘large’ conductive component like an IPG and closed when embedded in a poor conductor like human tissue. This eigenvector spectrum explains why currents induced on short (L ≲ 20 cm) implants have a tendency to appear in one specific pattern. Firstly, the relatively large first eigenvalue shows dominance of the first eigenvector. This is a reflection of the ability of an implant to support one current pattern better than another. The second reason is the likelihood of realistic RF exposures to match these modes. A large set of potential incident electric field distributions was used to show that the large majority of these potential incident fields projects most strongly on the first eigenmode. For all tested implants over 80% of electric field distributions excites the dominant mode more heavily (a number which increases up to 98% for 10 cm wires).

If, by coincidence, the second eigenmode is excited more heavily, the corresponding electric field distribution has an integral value far from the worst case (maximally 73%) found in the Duke model. Furthermore, the maximum of the induced current will not reach values close to the potential maximally induced current inside the Duke model (maximally 47%) which can be translated in an approximately four fold^11^ decrease in heating.

So, if strong heating is present due to an implant the current pattern causing it, will reflect the dominant eigenvector of its TM. Most severe heating will occur when the incident electric field projects predominantly onto this dominant eigenvector. These insights can be used to aid current monitoring techniques, identify hazardous situations and strategies^32^, ^33^, ^34^ to mitigate implant heating.

## CONFLICT OF INTERESTS

The authors have no conflicts to disclose.
